# Rare Case of Leukemoid Reaction in a Patient With Severe Alcoholic Hepatitis

**DOI:** 10.7759/cureus.9747

**Published:** 2020-08-14

**Authors:** Ted George O Achufusi, Anuj Sharma, Bishnu Sapkota, Kanish Mirchia

**Affiliations:** 1 Internal Medicine, State University of New York Upstate Medical University, Syracuse, USA; 2 Gastroenterology, State University of New York Upstate Medical University, Syracuse, USA; 3 Gastroenterology, Syracuse VA Medical Center, Syracuse, USA; 4 Pathology, State University of New York Upstate Medical University, Syracuse, USA

**Keywords:** alcoholic hepatitis, leukemoid reaction, leukocytosis, mallory denk bodies, dohle bodies

## Abstract

Alcoholic hepatitis results from excessive alcohol consumption in patients with or without underlying chronic liver disease. Leukemoid reactions have been associated with poor outcomes in severe alcoholic hepatitis. There are only a handful of reported cases describing this relationship, and the striking similarity in these cases was a high short-term mortality rate. We believe that these patients represent a unique subgroup of patients with alcoholic hepatitis and that leukemoid reaction is a poor prognostic indicator in this condition. Here, we describe a case of 55-year-old male with severe alcoholic hepatitis with superimposed candida esophagitis who was found to have leukemoid reaction during diagnostic workup.

## Introduction

Leukemoid reaction can be defined as persistent neutrophilic leukocytosis above 40,000 cells/UL caused by reactive causes outside of the bone marrow. It is characterized by a significant increase in the mature neutrophils and a differential count showing marked left shit [1]. This phenomenon can be seen in severe infections, malignancies, severe hemorrhage, or acute hemolysis. The exact cut-off value for leukocytes can vary, with lower and higher values also being used as a cut-off when describing leukemoid reactions. Leukemoid reaction is a rare occurrence in alcoholic hepatitis, with only a handful of case reports describing this occurrence. Here, we present the case of a 55-year-old male patient who presented to our institution with leukemoid reaction secondary to severe alcoholic hepatitis with superimposed candida esophagitis.

## Case presentation

**Figure 1:**
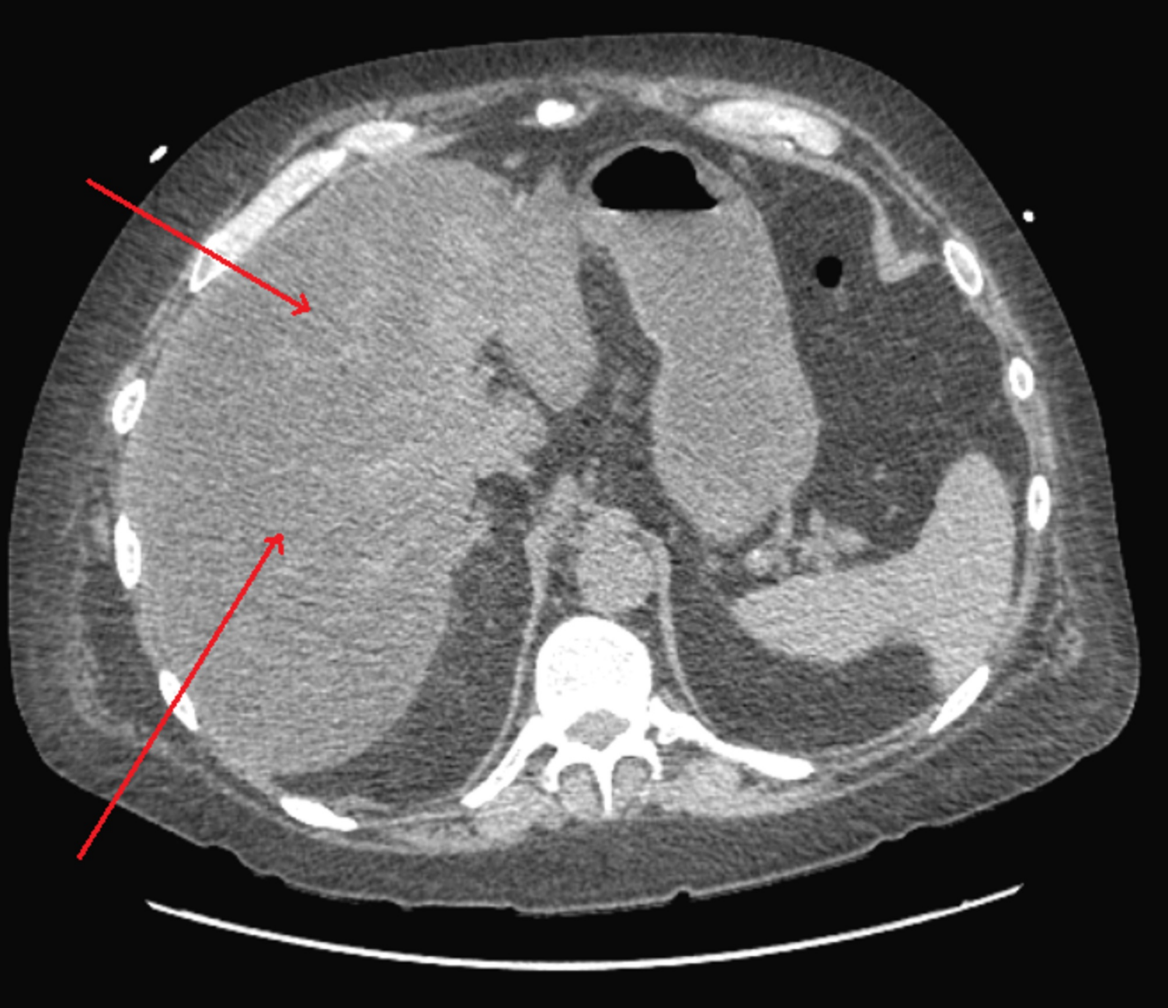
CT of the abdomen showing diffuse hepatic steatosis

A 55-year-old male with a past medical history of squamous cell carcinoma of the tongue, chronic kidney disease, gastroesophageal reflux disease, and severe alcohol use disorder presented to our institution for the evaluation of abnormal liver biochemistries and painless jaundice. The patient initially presented to the ED after suffering a fall at his home. Initial evaluation revealed diffuse jaundice. As such, lab work was obtained, which was significant for total bilirubin of 23.7 mg/dL (reference range: 0.1-1.2 mg/dL), direct bilirubin of 21 mg/dL (reference range: 0.1-0.3 mg/dL), alkaline phosphatase of 1,480 U/L (reference range: 44-147 IU/L), aspartate aminotransferase of 320 U/L (reference range: 5-40 IU/L), alanine aminotransferase of 180 U/L (reference range: 7-56 IU/L), white blood cell count of 40 x 10^9^/L (reference range: 4.5-11.0 × 10^9^/L), potassium of 5.4 mmol (reference range: 3.5-5 mmol/L), blood urea nitrogen of 51 mg/dL (reference range: 2.5-7.1 mmol/L), and creatinine of 5 mg/dL (reference range: 0.7-1.2 mg/dL). C-reactive protein was also elevated at 35 mg/dL (reference range: 0.1-1 mg/dL); however, lactic acid and procalcitonin were within normal limits at 2.3 mmol/L (reference range: 0.5-2.3 mmol/L) and 0.47 ng/mL (reference range: 0.10-0.49 ng/mL), respectively. Coagulation studies revealed an international normalized ratio of 1.2 and prothrombin time of 12 seconds (reference range: 11-13.5 seconds). The patient denied abdominal pain, fever, or chills, and vital signs were within normal limits. CT of the abdomen showed an enlarged liver and diffuse hepatic steatosis (Figure 1).

**Figure 2:**
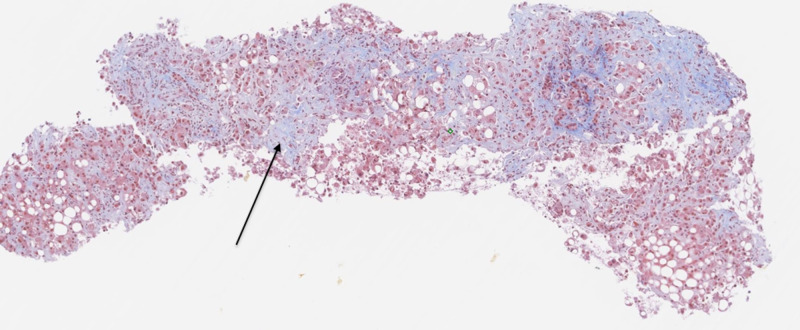
Liver biopsy specimen showing diffuse fibrosis

**Figure 3:**
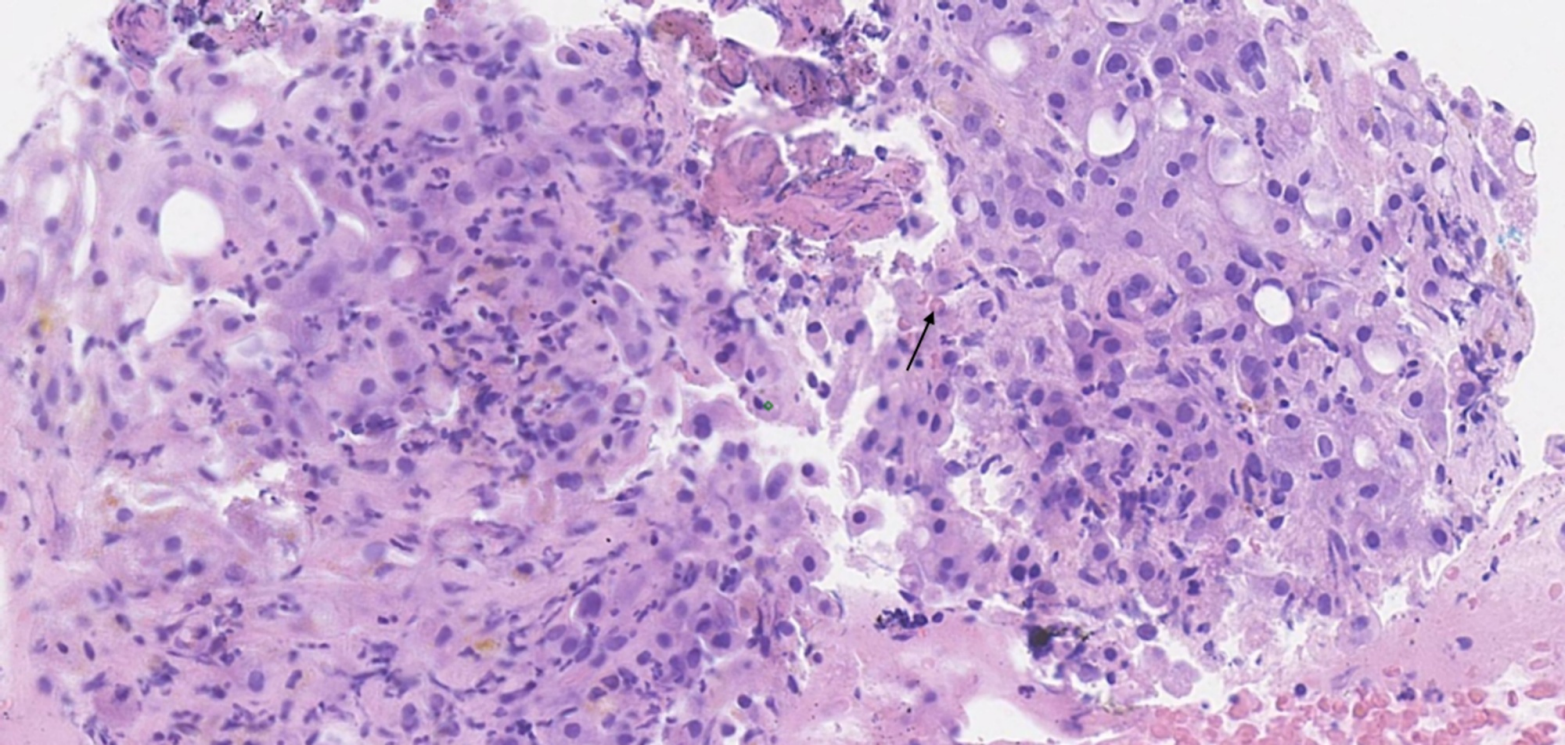
Neutrophilic inflammation and ballooning of hepatocytes along with Mallory-Denk bodies (black arrow)

On day one of admission, the patient was started on broad-spectrum antibiotics with cefepime and vancomycin. The etiology of his leukocytosis was unclear as extensive infectious workup was negative. Multiple sets of blood cultures did not show any growth, and there was no evidence of any abnormal collection on imaging. Urine analysis and X-ray chest were unremarkable. Despite the initiation of antibiotics, there was no improvement in white blood cell count, which peaked at 42 x 10^9^/L on the fifth day of admission. The gastroenterology service was consulted, who determined that the most likely etiology was alcoholic hepatitis in the setting of underlying cirrhosis. Given the negative infectious, metabolic, and autoimmune workup, the patient underwent endoscopic ultrasound (EUS)-guided liver biopsy, which showed diffuse fibrosis and neutrophilic infiltration (Figures 2, 3). The patient had an elevated Maddrey’s discriminant function score of 34, with an MELD (model for end-stage liver disease) score of 24.

**Figure 4:**
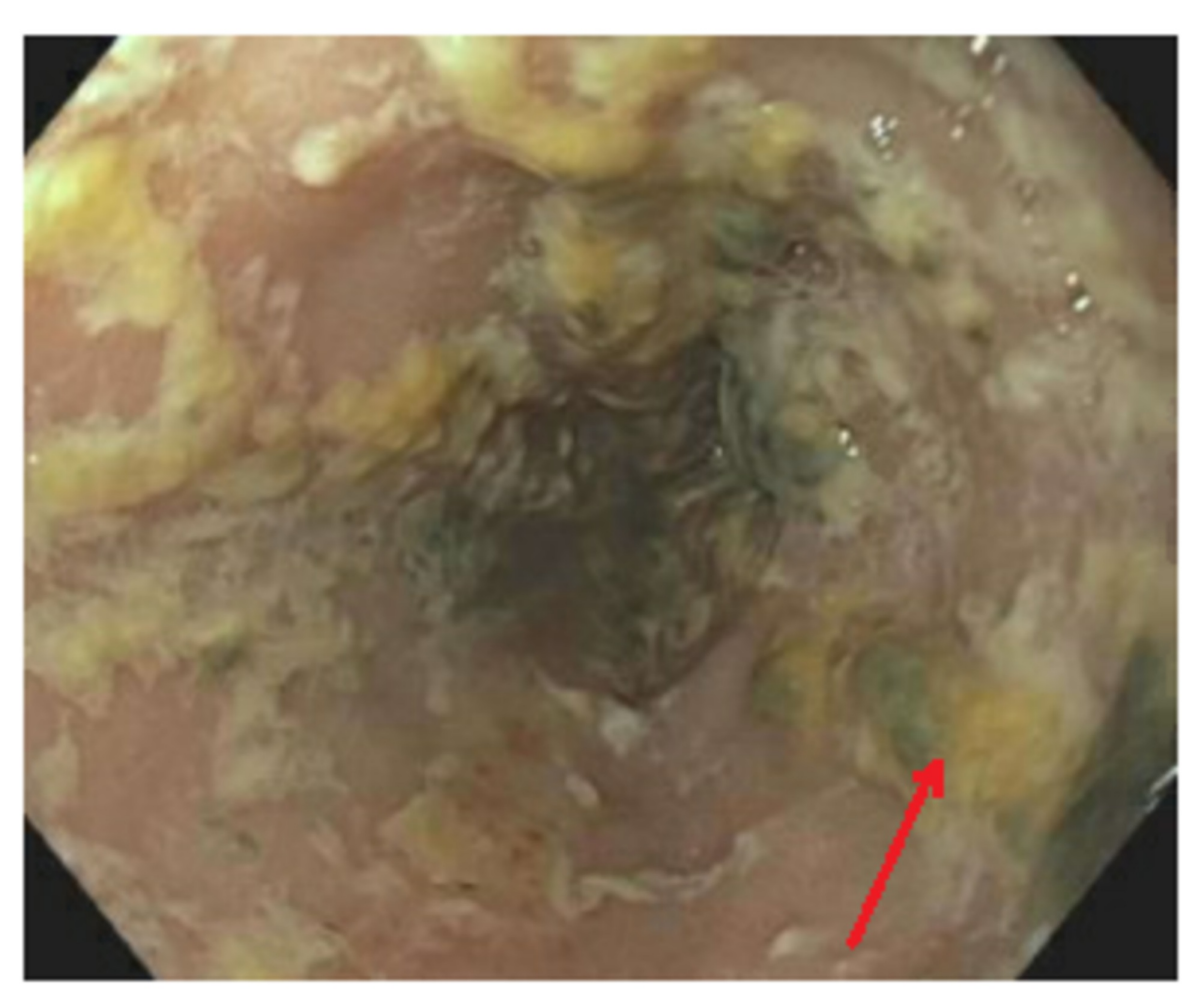
EGD showing esophageal candidiasis EGD, esophagogastroduodenoscopy

**Figure 5:**
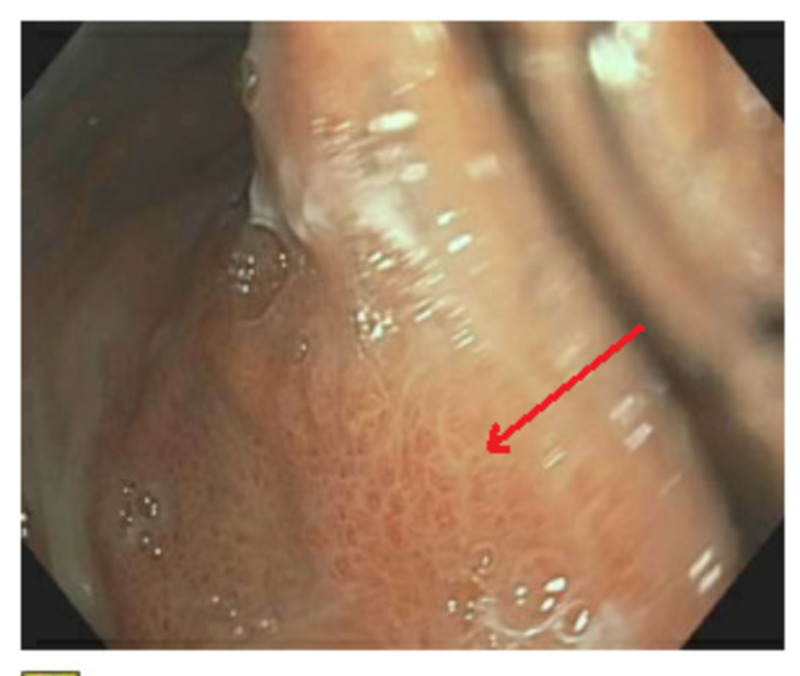
EGD showing "mosaic-like" pattern consistent with portal hypertensive gastropathy EGD, esophagogastroduodenoscopy

The patient was subsequently started on prednisolone for the treatment of alcoholic hepatitis, with only mild improvement in liver function tests. Total bilirubin peaked at 24.9 mg/dL and was at its lowest on the second day of prednisolone treatment at 20.3 mg/dL. In light of abnormal liver biochemistries, he underwent magnetic resonance cholangiopancreatography (MRCP), which was negative for biliary obstruction. Esophagogastroduodenoscopy (EGD) was significant for candida esophagitis, along with portal hypertensive gastropathy (Figures 4, 5). As such, steroids were discontinued. The patient was not a candidate for fluconazole given his liver injury and was started on micafungin which was later discontinued after learning of its potential hepatotoxicity. Because of the unusual presentation with such profound leukocytosis without evidence of sepsis, the hematology service was consulted to rule our possible hematologic malignancy. Bone marrow biopsy showed no evidence of myeloproliferative state. Janus kinase 2 (JAK 2) testing was negative, ruling out a myeloproliferative disorder. Unfortunately, the patient passed away on the 13th day of admission. Request for autopsy was declined; however, it was presumed that the patient expired as a result of alcoholic hepatitis.

## Discussion

Alcoholic hepatitis is a pro-inflammatory liver disease associated with short-term morbidity and mortality (25-35% in one month) [2]. The pathophysiology behind alcoholic hepatitis is attributed to oxidative stress, impairment of fatty acid oxidation, and generation of reactive oxygen species [3]. It has been hypothesized that pro-inflammatory cytokines are activated and secreted by Kupffer cells and other inflammatory cells following excessive alcohol exposure. Interleukin (IL)-6 and IL-8 are especially known for their chemotaxis activity, which may explain the high neutrophil presence in alcoholic hepatitis. Interestingly, the most distinctive histologic feature that differentiates alcoholic hepatitis from other forms of hepatitis is the predominantly neutrophilic inflammation (Figure 3). Other histological findings notable for alcoholic hepatitis include micro- and macrovesicular steatosis, hepatocellular necrosis, and Mallory-Denk bodies. Treatment of patients with severe alcoholic hepatitis, as defined by elevated Maddrey’s discriminant function, consists of a four-week course of either prednisolone or pentoxifylline. Data suggest corticosteroids have greater efficacy compared to pentoxifylline, although they are associated with higher risks of complications [4]. The challenge in this case was the inability to complete the course of steroids due to esophageal candidiasis, as esophageal infections and such a high MELD score are considered a contraindication for the use of corticosteroid therapy.

Hematologic abnormalities are rather common in moderate-to-severe alcoholic hepatitis, with moderate leukocytosis (<20,000/uL) a frequent finding in these cases [5]. It is rare, however, to encounter extremely high white blood cell counts in these cases, and the finding is associated with very high mortality rates. This case prompted us to review the literature describing this phenomenon. There were only a handful of case reports and case series describing a similar occurrence [6-8]. In a review of eight cases of patients with alcoholic hepatitis and associated leukemoid reaction, the white blood cell counts ranged from 57,000/uL to 129,000/uL. The striking similarity in all these cases was the high short-term mortality rate despite early initiation of treatment. It has previously been hypothesized that the pathophysiology behind this phenomenon may involve granulocyte colony-stimulating factor (G-CSF) released by damaged hepatocytes. Interestingly, experimental data has shown that administration of G-CSF improves survival among those with alcoholic hepatitis by inducing proliferation of hepatocytes within days of administration; however, additional studies are needed to know whether these findings translate into improved liver function [9].

**Figure 6:**
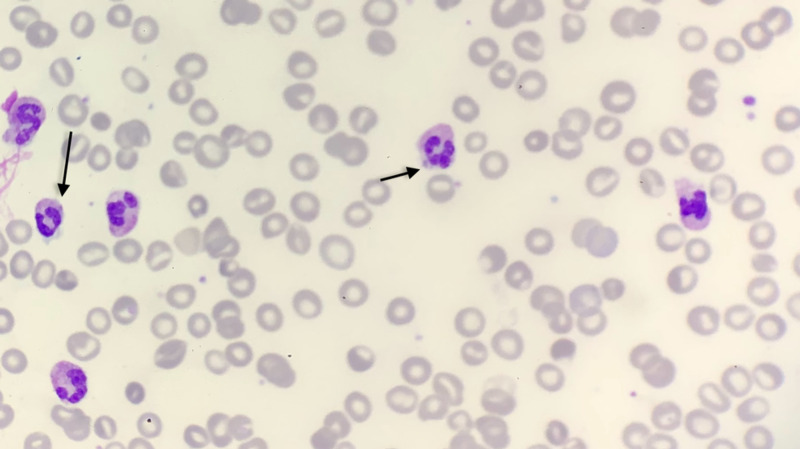
Peripheral smear showing Dohle bodies frequently seen in leukemoid reactions

The diagnostic approach in this group of patients involves exclusion of clonal disorders as well as testing for more obscures causes behind such drastic elevation in leukocyte count. Leukocyte alkaline phosphatase (LAP) score, serum vitamin B12 levels, a bone marrow biopsy, and blood smear, as well as serum levels of hemopoietic growth factors, are all helpful in distinguishing leukemoid reactions from other hematological disorders. In this case, the patient’s blood smear revealed toxic granulation and Dohle bodies, which are consistently present in leukemoid reactions (Figure 6) [10]. Full septic workup is also indicated in these situations. There was the initial concern for biliary obstruction and cholangitis in our patient due to elevated direct bilirubin levels; however, this was ruled out on diagnostic MRCP. The remainder of septic workup including multiple sets of blood cultures was also negative further raising suspicion for leukemoid reaction in the setting of alcoholic hepatitis. The absence of myeloproliferative state on bone marrow biopsy and peripheral blood smear findings of Dohle bodies further cemented the diagnosis.

## Conclusions

As seen in this case, high total leukocyte count in patients with alcoholic hepatitis is not always indicative of sepsis. Clinicians should consider alternative diagnoses including the possibility of underlying leukemoid reaction in patients with severe alcoholic hepatitis. This subgroup of patients should undergo a full diagnostic workup including a bone marrow biopsy to rule out hematologic malignancy. Corticosteroids should be considered for the initial course of treatment for severe alcoholic hepatitis as it has only shown benefit for short-term mortality.

## References

[1] Potasman I, Grupper M (2013). Leukemoid reaction: spectrum and prognosis of 173 adult patients. Clin Infect Dis.

[2] Singal AK, Kodali S, Vucovich LA, Darley-usmar V, Schiano TD (2016). Diagnosis and treatment of alcoholic hepatitis: a systematic review. Alcohol Clin Exp Res.

[3] Fung P, Pyrsopoulos N (2017). Emerging concepts in alcoholic hepatitis. World J Hepatol.

[4] Liang R, Liu A, Perumpail RB, Wong RJ, Ahmed A (2015). Advances in alcoholic liver disease: an update on alcoholic hepatitis. World J Gastroenterol.

[5] Stewart S, Prince M, Bassendine M (2007). A randomized trial of antioxidant therapy alone or with corticosteroids in acute alcoholic hepatitis. J Hepatol.

[6] Morales AM, Hashimoto LA, Mokhtee D (2006). Alcoholic hepatitis with leukemoid reaction after surgery. J Gastrointest Surg.

[7] Argüelles-Grande C, Leon F, Matilla J, Domínguez J, Montero J (2002). Steroidal management and serum cytokine profile of a case of alcoholic hepatitis with leukemoid reaction. Scand J Gastroenterol.

[8] Juturi JV, Hopkins T, Farhangi M (1998). Severe leukocytosis with neutrophilia (leukemoid reaction) in alcoholic steatohepatitis. Am J Gastroenterol.

[9] Spahr L, Lambert JF, Rubbia-Brandt L (2008). Granulocyte-colony stimulating factor induces proliferation of hepatic progenitors in alcoholic steatohepatitis: a randomized trial. Hepatology.

[10] Mitchell RG, Michael M, Sandidge D (1991). High mortality among patients with the leukemoid reaction and alcoholic hepatitis. South Med J.

